# Protective effects of resveratrol against mancozeb induced apoptosis damage in mouse oocytes

**DOI:** 10.18632/oncotarget.14056

**Published:** 2016-12-21

**Authors:** Yu Liu, Ya-Long Wang, Shu-wen He, Ming-Huang Chen, Zhen Zhang, Xian-Pei Fu, Bin-Bin Fu, Bao-Qiong Liao, Yan-Hong Lin, Zhong-Quan Qi, Hai-Long Wang

**Affiliations:** ^1^ Organ Transplantation Institute, Medical College, Xiamen University, Xiamen City, Fujian Province, China; ^2^ Fujian Key Laboratory of Organ and Tissue Regeneration, Xiamen City, Fujian Province, China; ^3^ Department of Gynaecology and Obstetrics, Zhongshan Hospital, Xiamen University, Xiamen City, Fujian Province, China; ^4^ Xiamen Institute for Food and Drug Quality Control, Xiamen City, Fujian Province, China; ^5^ Department of Gynecology, The First Affiliated Hospital of Fujian Medical University, Fuzhou, China

**Keywords:** oocyte, mancozeb, resveratrol, apoptosis

## Abstract

Mancozeb, a mixture of ethylene-bis-dithiocarbamate manganese and zinc salts, is one of the most widely used fungicides in agriculture. Mancozeb could lead to mitochondria dysfunction, cellular anti-oxidation enzymes depletion and apoptotic pathways activation. Previous studies indicated the exposure of mancozeb through mother would lead to irregular estrous cycles, decreased progesterone levels, reduced litter sizes, and more frequent delivery of dead fetuses. In this study, we investigated mancozeb inducing reproductive toxicity, especially focusing on its apoptotic effect and epigenetic modifications. We also showed that resveratrol, a kind of phytoalexin found in peanuts and grapes, can alleviate mancozeb's adverse effects, such as declined fertility, decreased ovary weight and primary follicles. Besides, mancozeb treated oocytes displayed suboptimal developmental competence and this can also be improved by treatment of resveratrol. More detailed investigation of these processes revealed that mancozeb increased reactive oxygen species, causing cell apoptosis and abnormal epigenetic modifications, and resveratrol can block these cytotoxic changes. Collectively, our results showed that resveratrol can alleviate mancozeb induced infertility and this was mainly through the correction of apoptotic tendency and the abnormity of cellular epigenetic modification.

## INTRODUCTION

Mancozeb, a manganese/zinc ethylene-bis-dithiocarbamate, is a pesticide routinely used in pest control management. It acts as a contact fungicide used to protect vegetables, fruits and other field crops against fungal diseases [1]. Besides, it has a wide application on residential lawns, golf courses and agricultural lands over the United States (USGS; www.usgs.gov). Human exposure to mancozeb may occur through residues present in food and drinking water [2, 3]. Mancozeb mainly targets mitochondrial enzymes, disturbing mitochondrial function and adenosine triphosphate (ATP) production. In addition, it can react with the sulfhydryl groups of amino acids and enzymes in fungal cells, resulting in metabolism disorders and activated apoptotic pathways [4]. Although it has been reported to possess low acute toxicity and scarce persistence in the environment, diverse chronic adverse health effects of mancozeb have been reported, such as increased carcinogenic potential [5], endocrine system disruption, and toxic effects on the immune [6] and neuronal system [7].

Furthermore, mancozeb could affects female fertility either by targeting the hypothalamo-hypophysial-ovarian axis [8] or inducing a direct cytotoxicity on oocytes [9, 10], embryo [11] and granulosa cells [12]. A previous report has shown a strong relationship between mancozeb exposure and increased incidence of thyroid disease in female spouses of pesticide applicators. Additionally, a Norwegian research associated mancozeb exposure with neural tube defects in newborns from farmer families [13]. Other effects on reproductive system organs and functions have been observed including decreased uterus, ovary, and testes weights, disrupted estrus cycles, and pathological changes in the reproductive organs [8, 14, 15]. As regards mancozeb cytotoxic effects, previous research studies suggested that it mancozeb may induce a reduction of mouse fertilization rate together with alterations of oocyte meiotic spindle morphology, impaired mouse embryo development and blastomere apoptosis induction [10, 16]. Moreover, mancozeb induces dose-dependent morphological modifications and substantial alteration of p53 pathway in both mouse and human granulosa cells [12]. These previous studies supported that mancozeb is toxic to fertility, but most of these studies were based on *in vitro* or acute exposure observation and the mechanisms of toxicity are still not fully understood. In the current study, we investigated the effect of mancozeb on female ovaries and oocytes through apoptosis and epigenetic modification aspects with the mouse model, to find the mechanism and groundwork for a strategy regarding to chronic exposure of this dithiocarbamate.

Resveratrol (3, 5, 4′-trihydroxystilbene), a phytoalexin molecule belonging to stilbene family, is produced in a variety of plant species, particularly in peanuts and grapes. Mounting evidences are emphazing resveratrol wide variety of bio-properties, including its anti-inflammatory [17, 18], cardio-protective [19], anti-cancer [20, 21], anti-microbial [22], and anti-aging effects [23]. These biological activities are regulated by a These biological activities are regulated carried out by a broad mechanisms diversity, most explored one and thus well-known is its anti-oxidant activity due to free radical scavenging [24]. This effect can be illustrated as best by its well-established effect on atherosclerosis. Moreover, resveratrol functions as a potent SIRT activator which is a key factor in caloric restriction, thus increasing the lifespan in a variety of organisms [25]. Resveratrol also functions as a phytoestrogen, regulating the reproductive systems by changing the level of estrogen through estrogen receptors (ERs) binding, increasing progesterone secretion as well as mRNA levels of Sirt1, LH receptor, and steroidogenic regulatory protein in cells [26, 27]. More notably, resveratrol could develop a powerful anti-apoptotic effect in organisms through its action on several different pathways including interleukin family [28], p53 signal [29], and ROS-dependent pathways [30].

We hypothesized that supplement of resveratrol could counteract mancozed induced infertility and performed a long-term administration of mancozed and resveratrol in mice to test apoptosis relative effects of these two agents. We investigated fetal outcomes, histopathological changes of ovaries, oocyte maturation competence, apoptosis of oocyte and granulosa cells, and epigenetic alteration of oocytes. Our results showed that resveratrol could alleviate the adverse effect of mancozed on female reproductive systems.

## RESULTS

### Resveratrol counteracts mancozeb induced fertility decline and increases primary follicles

**Figure 1:**
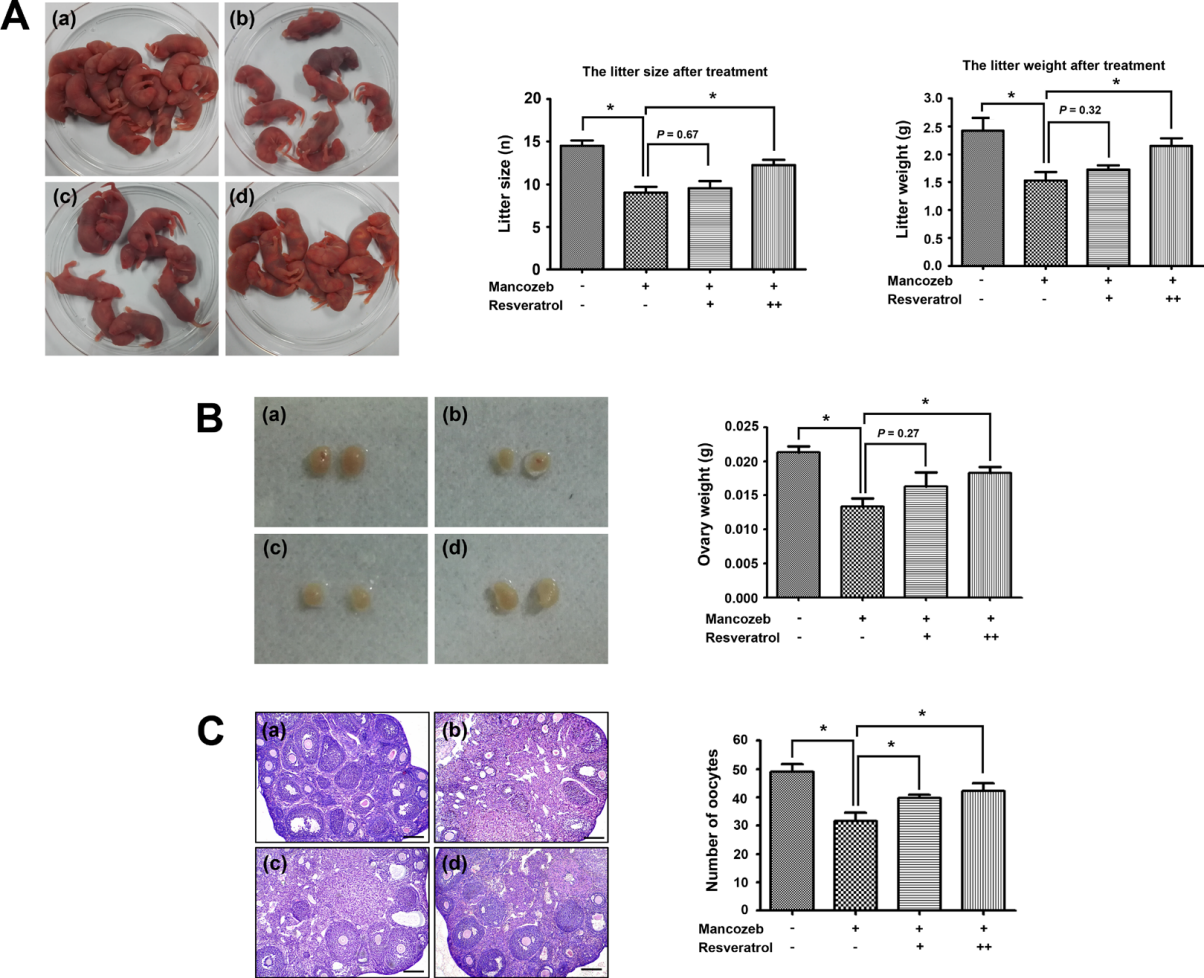
Effect of resveratrol on mancozeb reduced fertility (**A**) Mancozeb treated mice showed a significantly reduced average offspring. Resveratrol treatment could protect against mancozeb effect, acting on both litter size and litter weight. (**B**) Ovaries in mancozeb treated mice were also significantly smaller when compared with those of controls. Resveratrol could protect against mancozeb inducing ovary weight reduction. (**C**) Primary and growing follicles were located at the ovarian cortex in the control group, whereas more atretic follicles and less primary follicles were present in mancozeb groups. Growing and mature follicles were also present in the resveratrol treated group. Oocytes number from different groups were calculated. *P < 0.05. Scale bar = 200 μm. For each individual result, (a) control group; (b) mancozeb treated group; (c) resveratrol (Low) group and (d) resveratrol (High) group.

After 4 weeks administration, mice in each group did not show any difference in weight, as well as no abnormal behaviors. We first followed the effect of resveratrol and mancozeb on reproductive behaviors and the litters were observed in successfully mated females. Mancozeb treated mice generated significantly reduced litter size (9.00 ± 0.70, *n* = 8, *P* < 0.05 versus control group: 14.50 ± 0.64, *n* = 8) and litter weight (1.53 ± 0.14 g, *n* = 28, *P* < 0.05 versus control group: 2.42 ± 0.23 g, *n* = 44) compared with that of control group. 100 mg/mL resveratrol has no protective effects regarding to the both aspects (as to litter size: 9.50 ± 0.86, *n* = 8, *P* > 0.05 versus mancozeb group; as to litter weight: 1.72 ± 0.08 g, *n* = 29), but a relative high level of resveratrol (200 mg/mL) could significantly improve the litter situation (as to litter size: 12.25 ± 0.63, *n* = 8, *P* < 0.05 versus mancozeb group; as to litter weight: 2.15 ± 0.14 g, *n* = 37, *P* < 0.05 versus mancozeb group) (Figure 1A).

In addition, we found resveratrol could protect from mancozeb induced ovary weight decreased. The ovary weights in different groups were the followings: 213.3 ± 8.8 mg for control group (*P* < 0.05 versus mancozeb group, *n* = 6), 133.3 ± 12.0 mg for mancozeb group (*n* = 6), 163.3 ± 20.3 mg for resveratrol (Low) group (*P* < 0.05 versus mancozeb group, *n* = 6) and 183.3 ± 8.8 mg for resveratrol (High) group (*P* < 0.05 versus mancozeb group, *n* = 6) (Figure 1B). HE staining results showed in a normal ovarian architecture and morphology with regular follicles in the control group. Nevertheless, the number of atretic follicles increased, while the number of normal oocytes decreased in the mancozeb treated group, compared to the control group (31.7 ± 2.9, *P* < 0.05 versus control group: 49.0 ± 2.6). However, resveratrol exerted a clear protective effect against mancozeb toxicity, since ovary in the resveratrol treatment group showed a decreased number of atretic follicles, increased number of growing and mature follicles, and increased number of oocytes (Low: 39.7 ± 1.3, *P* < 0.05 versus mancozeb group and High: 42.3 ± 2.6, *P* < 0.05 versus mancozeb group) (Figure 1C).

### Effects of resveratrol and mancozeb on the oocytes quality and development potential

**Figure 2:**
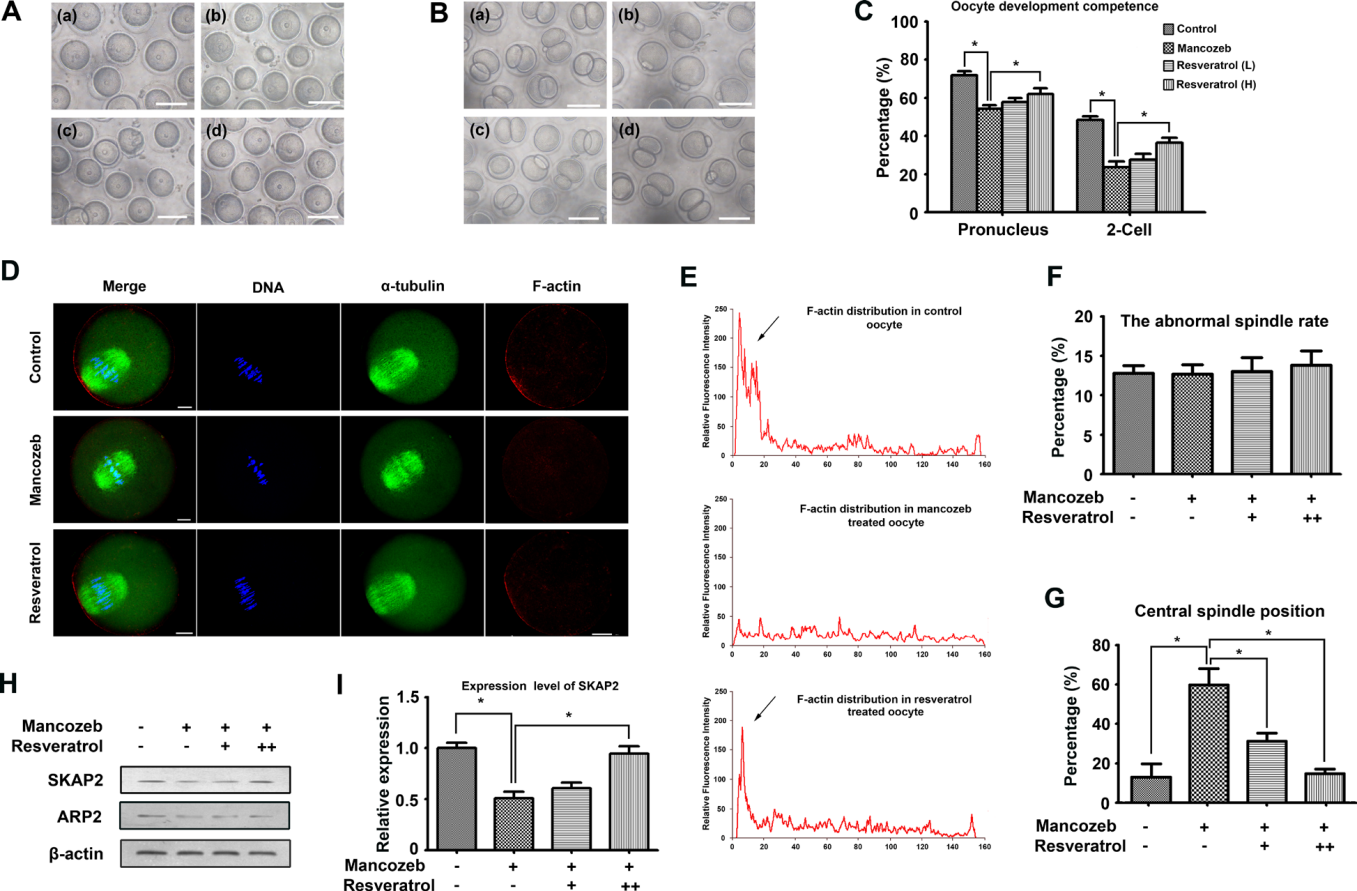
Effects of resveratrol on mancozeb affecting oocytes quality and developmental competence (**A**) GV oocytes morphology from mice ovaries in different groups. Scale bar =100 μm. (**B**) Two-cell stage embryo morphology after parthenogenetic activation. Resveratrol treated oocytes exhibited a higher potential to reach the two-cell stage than that mancozeb treated, statistical analysis are showed in (**C**), Scale bar =100 μm. (**D**) Actin cap assembled at the cortex in normal oocytes but dispersed after mancozeb treatment. Recurrence of actin cap was observed after resveratrol treatment. Spindle did not show any difference in each group. Green, α-tubulin; Red, actin; Blue, DAPI staining of DNA. Scale bar = 20 μm. (**E**) Fluorescent intensity of F-actin in different oocyte areas in different groups. Arrows are zones of actin cap. (**F**) Percentage of oocytes with abnormal spindle formation in different groups. (**G**) Percentage of oocytes with centrally localized spindles in different groups. (**H**) Western blot of Arp2 and Skap2 protein levels in oocytes after different treatment. (**I**) Gray intensity of Skap2 in different groups.

GV oocytes were collectd from the ovaries and cultured in M2 medium. As shown in Figure 2A, most of the oocytes from control and resveratrol group had a clear cytoplasm, smooth zona pellucida and normal morphology. However, oocytes from mancozeb-treated mice witnessed a shrunk and dark cytoplasm or a coarse zona pellucida. After 12 h culture, most oocytes from all mice group extruded the first polar body. No clear difference was observed regarding the rate of polar body extrusion. However, when oocytes underwent parthenogenetic activation, the rate of pronucleus and two-cell formation in the mancozeb treated group significantly decreased (pronucleus: 54.46% ± 1.85, *P* < 0.05 versus control group, *n* = 187; 2-cell: 22.87% ± 3.02, *P* < 0.05 versus control group, *n* = 144), which in control (pronucleus: 72.01% ± 2.18, *n* = 182; 2-cell: 46.77 % ± 1.89, *n* = 145) and resveratrol group is higher (pronucleus: 58.00 % ± 2.12 (Low) (*n* = 195) and 62.17 % ± 3.06 (High) (*n* = 171), *P* < 0.05 versus mancozeb group; 2-cell: 26.60% ± 3.00 (low) (*n* = 165) and 35.30% ± 2.43 (high) (*n* = 147), *P* < 0.05 versus mancozeb group) (Figure 2B, 2C).

To understand resveratrol and mancozeb mechanism of action on oocyte development, we followed cytoskeleton changes in different group. We first evaluated meiotic spindle organization in mouse oocytes. The results revealed that mancozeb did not affect spindle formation. Both mancozeb and resveratrol treated oocytes exhibited optimal spindle morphologies and well-aligned chromosomes (Figure 2D, 2F). However, mancozeb affected actin expression. Actin cap formatted in oocyte from control group dismissed in the mancozeb treated oocytes but works well in the resveratrol group. In addition, the actin guided asymmetric spindle positioning showed differences (Figure 2D, 2E). Those oocytes with central spindle increased from 10.18% ± 3.57 (*n* = 62) in control group to 61.23% ± 8.26 (*n* = 72) in mancozeb treated group (*P* < 0.05 versus control group), but still stay at 32.03% ± 4.14 (*n* = 68, *P* < 0.05 versus mancozeb group) in the resveratrol (Low) group and 15.13% ± 2.47 (*n* = 72, *P* < 0.05 versus mancozeb group) in the resveratrol (High) group (Figure 2D, 2G).

We also investigated actin nucleation factor Arp2 and Skap2 protein expression. Western blot analysis revealed that Arp2 and Skap2 protein expression were significantly reduced after mancozeb treatment when compared with the expression in control group. Resveratrol had no effects on Arp2, but obviously increased the Skap2 level (Figure 2H, 2I).

### Resveratrol alleviates mancozeb induced ROS-mediated apoptosis in ovaries and oocytes

**Figure 3:**
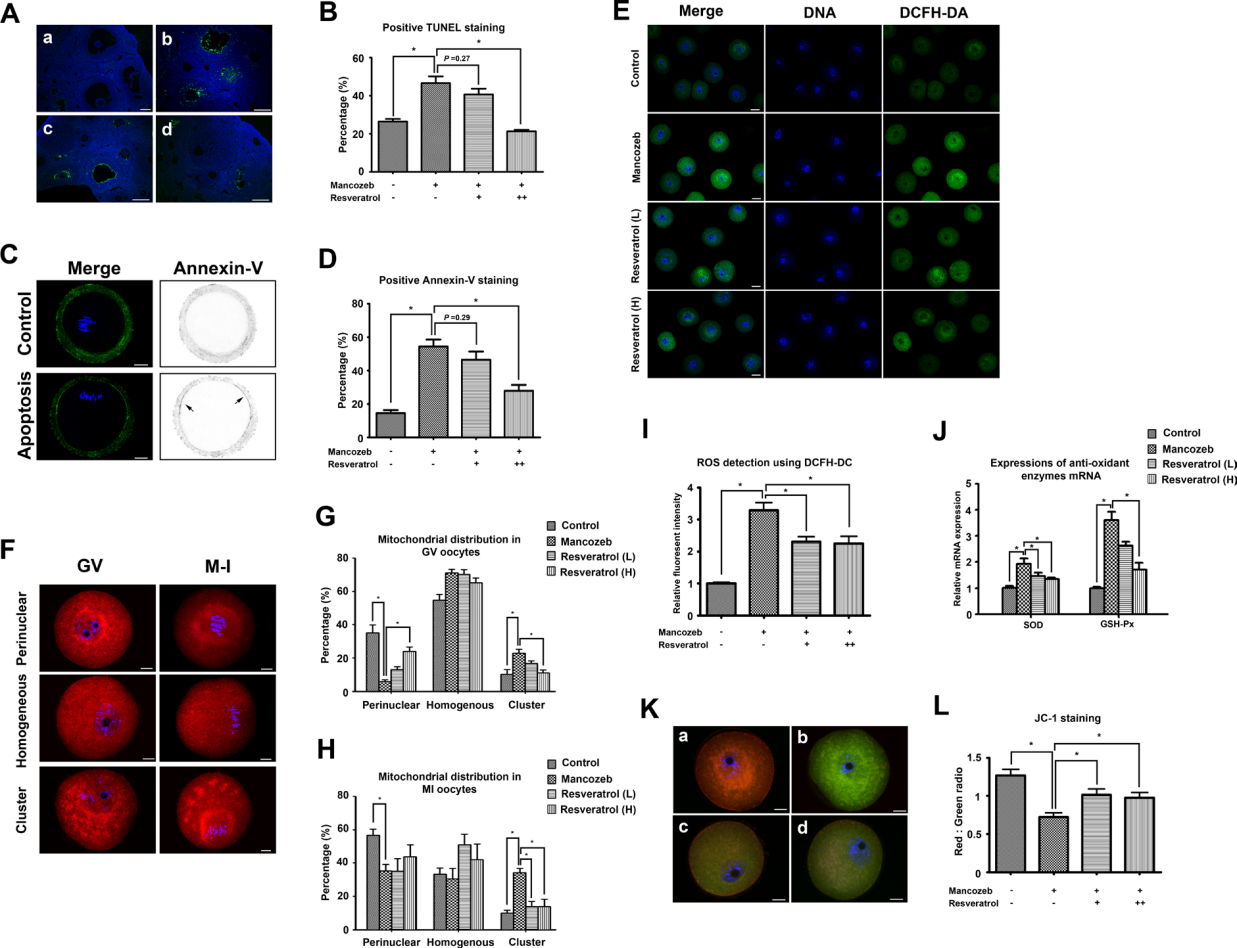
Effect of resveratrol on mancozeb induced ROS related apoptosis in ovary and oocyte (**A**) Immunofluorescence images of terminal deoxynucleotidyl transferase-mediated dUTP nick-end labeling (TUNEL) (green) in ovaries. DNA stained with DAPI (blue). Scale bar = 100 μm (**B**) Percentage of follicles with positive TUNEL staining in different groups. (**C**) Immunofluorescence images of Annexin-V (green) in oocytes. Control oocytes exhibited fluorescence only in the zona pellucida, but early apoptositic oocytes exhibited fluorescence in both zona pellucida and membranes. Scale bar = 20 μm. (**D**) Percentage of oocytes with positive Annexin-V staining in different groups. (**E**) Detection of ROS levels using DCFH-DA probe. GV oocytes were cultured in M2 medium plus 0.1% DCFH-DA for 30 min. The fluorescence of the hydrolyzed product DCF (green) reflects ROS levels. Scale bar = 100 μm. (**F**) Effects of mancozeb and resveratrol on the distribution of mitochondria in meiosis. MitoTracker Red staining showed a perinuclear pattern of mitochondria in most of the control oocytes. This pattern was modified by mancozeb and resveratrol treatment. Scale bar = 20 μm. Statistical analysis of different mitochondrial distribution patterns in GV oocytes and MI oocytes in different groups are listed in (**G**) and (**H**). (**I**) ROS levels quantified from DCF fluorescence in (E) in different groups. (**J**) Anti-oxidant enzyme SOD and GSH-Px mRNA levels in oocytes from each group. (**K**) JC-1-stained mitochondria in GV oocytes. Mitochondria with high membrane potential showed a red fluorescence while those with low membrane potential showed a green fluorescence. Scale bar = 20 μm. (**L**) Red-green JC-1 fluorescence ratio in oocytes from different groups. (a) control group; (b) mancozeb treated group; (c) resveratrol (Low) group and (d) resveratrol (High) group. *P < 0.05.

To illustrate the deep mechanism how resveratrol and mancozeb affect fertility, we analyzed the apoptotic rate in both ovaries and oocytes. As shown in Figure 3A, fluorescence-mediated TUNEL staining in the mancozeb group highlighted increasing number of apoptotic granulosa cells around the growing follicless, as compared with the control group. However, resveratrol treatment attenuated the amount of TUNEL-positive granulosa cells in the secondary follicles. The percentages of positive TUNEL follicle in different groups were the following: 26.43 ± 1.28% (*n* = 74) in control group, 46.53% ± 3.55 in the mancozeb group (*n* = 64, *P* < 0.05 versus control group), 40.67% ± 2.96 in the resveratrol (Low) group (*n* = 63, *P* > 0.05 versus mancozeb group) and 21.37% ± 0.78 in the resveratrol (High) group (*n* = 65, *P* < 0.05 versus mancozeb group) (Figure 3B).

We next used Annexin-V staining to study whether apoptosis occurred in oocytes. As shown in Figure 3C, FITC conjugated Annexin-V only existed in the control oocytes was only present in at the zona pellucida, indicating non-apoptosis. In contrast, clear green fluorescence was found in the membrane and zona pellucida of the mouse oocytes after mancozeb treatment, suggesting the occurrence of early apoptosis. Resveratrol could alleviate mancozeb induced apoptosis, as shown by the absence of green fluorescence in the membrane. The percentages of Annexin-V positive oocytes in different groups were the following: 26.43% ± 1.28 (*n* = 82) in the control group, 46.53% ± 3.55 in the mancozeb group (*n* = 81, *P* < 0.05 versus control group), 40.67% ± 2.96 in the resveratrol (Low) group (*n* = 82, *P* > 0.05 versus mancozeb group) and 21.37% ± 0.78 in the resveratrol (High) group (*n* = 72, *P* < 0.05 versus mancozeb group) (Figure 3C, 3D).

Impaired ROS balance is considered the main factor leading to apoptosis. Thus, we evaluated ROS level as well as some enzyme activity involving in ROS metabolism. As shown in Figure 3E, the mancozeb treated oocytes showed a high DEFH-DA positive fluorescence, corresponding to an increased level of ROS. This accumulation of ROS could be prevented by resveratrol, as shown by the decreased DEFH-DA fluorescence intensity from 3.29 ± 0.24 in the mancozeb group (*n* = 15, *P* < 0.05 versus control group: 1.00 ± 0.03) to 2.31 ± 0.15 in the resveratrol (Low) group (*n* = 15, *P* < 0.05 versus mancozeb group) and 2.24 ± 0.23 in the resveratrol (High) group (*n* = 15, *P* < 0.05 versus mancozeb group) (Figure 3I). We also measured the change in the expression of antioxidant enzymes required for radicals’ detoxification. As shown in Figure 3J, resveratrol effectively decreased the up-regulated SOD expressions induced by mancozeb (control: 1.00 ± 0.08; mancozeb: 1.94 ± 0.19, *P* < 0.05 versus control group; resveratrol (Low): 1.46 ± 0.13, *P* < 0.05 versus mancozeb group; resveratrol (High): 1.35 ± 0.06, *P* < 0.05 versus mancozeb group). Furthermore, the same tendency was observed in the alterations of GSH-Px in different groups (control: 1.00 ± 0.06; mancozeb: 3.60 ± 0.32, *P* < 0.05 versus control group; resveratrol (Low): 2.62 ± 0.15, *P* > 0.05 versus mancozeb group; resveratrol (High): 1.70 ± 0.26, *P* < 0.05 versus mancozeb group).

Mitochondria play an important role in the cellular processes of both ROS metabolism and apoptosis. Thus, we investigated mitochondrial distribution in oocytes after different treatments. As shown in Figure 3F, 3G, 3H, most of the normal GV oocytes had a perinuclear or homogenous mitochondrial distribution and metaphase I (MI) oocytes had a spindle-surrounding distribution. In contrast, mancozeb led to an abnormal mitochondrial distribution. Mitochondrial clusters were also present in the cytoplasm, suggesting mitochondria in their suboptimal status.

The mitochondrial distribution patterns rates in the control GV oocytes were the following: perinuclear (35.10% ± 4.70), homogenous (54.67% ± 3.53) and cluster (10.23% ± 2.91). By comparison, for mancozeb treated oocyte, the rates of mitochondrial distribution patterns were: perinuclear (6.03% ± 1.03), homogenous (70.90 % ± 2.23) and cluster (22.97% ± 2.34). This effect could be blocked by resveratrol, since the resveratrol treated oocytes showed mitochondrial distribution patterns as follows: perinuclear (13.10% ± 1.73 (Low); 23.83% ± 2.80 (High)), homogenous (70.83% ± 2.81 (Low); 65.03 % ± 2.85 (High)) and cluster (16.77 % ± 1.69 (Low); 11.13% ± 1.82 (High)) (*n* = 80, control group; *n* = 82, mancozeb group; *n* = 85, resveratrol (Low) group; *n* = 80, resveratrol (High) group).

The mitochondrial distribution rates in the control MI oocytes were the following: perinuclear (56.57% ± 3.75), homogenous (33.37% ± 3.74) and cluster (10.10% ± 1.74). By comparison, for mancozeb treated oocyte, the rates of mitochondrial distribution patterns were: perinuclear (35.27 % ± 3.96), homogenous (30.50 % ± 6.35) and cluster (34.20 % ± 2.72). The same protective effect was observed after resveratrol treatment, since the rates of mitochondrial distribution patterns were the following: perinuclear (35.13 % ± 7.60 (Low); 43.83 % ± 7.06 (High)), homogenous (50.90 % ± 6.49 (Low); 42.17 % ± 9.24 (High)) and cluster (13.97 % ± 2.99 (Low); 14.00 % ± 4.22 (High)) (*n* = 89, control group; *n* = 85, mancozeb group; *n* = 84, resveratrol (Low) group; *n* = 91, resveratrol (High) group).

We also investigated the mitochondrial membrane potential using JC-1 as a proxy. As shown in Figure 3K, control oocytes possessed a polarized mitochondrial membrane potential, as indicated by the strong red JC-1 emission, whereas mancozeb treatment diminished red in favor of green JC-1 emission (Figure 3K and 3L). The ratio of red to green JC-1 fluorescence, an indicator of the polarized degree, decreased from 1.27 ± 0.08 (*n* = 15) in control oocytes to 0.72 ± 0.06 (*n* = 15, *P* < 0.05 versus control group) in mancozeb oocytes. However, resveratrol partially protect from the effect of mancozed, since red – green ratio JC-1 fluorescence decreased to 1.01 ± 0.08 (*n* = 15, *P* < 0.05 versus mancozeb group) in resveratrol (Low) and 0.97 ± 0.07 (*n* = 15, *P* < 0.05 versus mancozeb group) in resveratrol (High) group.

### Effects of mancozeb and resveratrol on the epigenetic alterations in mouse oocytes

**Figure 4:**
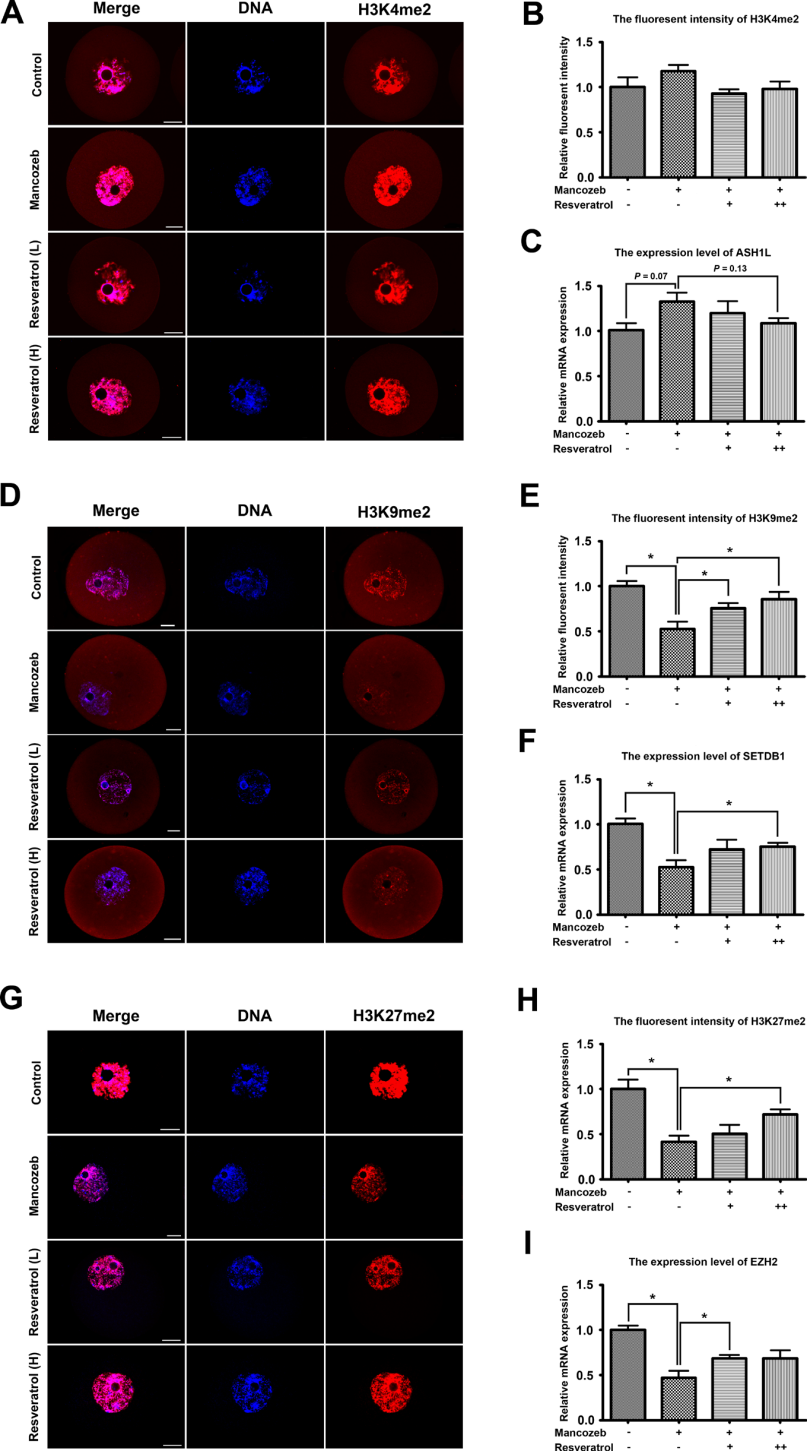
Resveratrol and mancozeb effected on H3K4me2, H3K9me2 and H3K27me3 levels in mouse oocytes (**A**) Immunofluorescence images of H3K4me2 in oocytes after different treatments. Red, H3K4me2; Blue, DAPI staining of DNA. Scale bar = 20 μm. (**B**) Average H3K4me2 fluorescence intensity in oocytes. (**C**) ASH1L mRNA levels in oocytes from each group. (**D**) Immunofluorescence images of H3K9me2 in oocytes after different treatment. Red, H3K9me2; Blue, DAPI staining of DNA. Scale bar = 20 μm. (**E**) Average H3K9me2 fluorescence intensity in oocytes. (**F**) SETDB1 mRNA levels in oocytes from each group. (**G**) Immunofluorescence images of H3K27me3 in oocytes after different treatment. Red, H3K27me3; Blue, DAPI staining of DNA. Scale bar = 20 μm. (**H**) Average H3K27me3 fluorescence intensity in oocytes. (**I**) EZH2 mRNA levels in oocytes from each group.

Next, we investigated whether mancozeb and resveratrol exerted effects on the epigenetic modifications in mouse GV oocytes. We first examined the fluorescent intensity of H3K4me2, H3K9me2 and H3K27me3 in each group. As shown in Figure 4A, H3K4me2 was co-localized with DNA, but comparing with controls, the levels of H3K4me2 methylation with mancozeb treatment showed no alterations. Statistical analysis in Figure 4B displayed the relative fluorescent intensity increased from 1.00 ± 0.10 (*n* = 15) in control group to 1.18 ± 0.07 (*n* = 15) in mancozeb group. Conversely, H3K9me2 in mancozeb group was significantly reduced (0.52 ± 0.08, *P* < 0.05 versus control group, *n* = 15) when compared with that in the control (1.00 ± 0.06, *n* = 15) (Figure 4D and 4E). We also examine H3K27me3 and we found the same trend of methylated modification as H3K9me2 (0.41 ± 0.07, *n* = 15 in mancozeb group versus 1.00 ± 0.10, *n* = 15 in control group, *P* < 0.05 versus control group) (Figure 4G and 4H). Beside the modifications of lysine residue H3K4me2, resveratrol could narrow the gap between mancozeb and control group, with the evidence of changed fluorescence intensity in Figure 4B, and 4H. For H3K4me2, the fluorescence intensities are 0.93 ± 0.05 in the resveratrol (Low) group and 0.98 ± 0.08 in the resveratrol (High) group (both *n* = 15). For H3K9me2, the fluorescence intensities are 0.76 ± 0.05 (*P* < 0.05 versus mancozeb group) in the resveratrol (Low) group and 0.85 ± 0.08 (*P* < 0.05 versus mancozeb group) in the resveratrol (High) group (both *n* = 15). For H3K27me2, the fluorescence intensities are 0.51 ± 0.10 (*P* > 0.05 versus mancozeb group) in the resveratrol (Low) group and 0.72 ± 0.06 (*P* < 0.05 versus mancozeb group) in the resveratrol (High) group (both *n* = 15). Subsequently, we examined mRNA expression levels of H3K4me2 methyltransferase ASH1L, H3K9me2 methyltransferase SETDB1 and H3K27me3 methyltransferase EZH2. As shown in Figure 4C, and 4I, after mancozeb treatment, the mRNA expression of SETDB1 and EZH2 significantly increased, but not obviously regarding ASH1L. The altered mRNA expressions in resveratrol group showed a same trend as the changed fluorescence intensity respectively (For ASH1L, control: 1.00 ± 0.08; mancozeb: 1.29 ± 0.10 (*P* > 0.05 versus control group); resveratrol (Low): 1.20 ± 0.13 (*P* > 0.05 versus mancozeb group); resveratrol (High): 1.08 ± 0.06 (*P* > 0.05 versus mancozeb group). For SETDB1, control: 1.00 ± 0.06; mancozeb: 0.52 ± 0.08 (*P* < 0.05 versus control group); resveratrol (Low): 0.72 ± 0.10 (*P* > 0.05 versus mancozeb group); resveratrol (High): 0.75 ± 0.04 (*P* < 0.05 versus mancozeb group). For EZH2, control: 1.00 ± 0.04; mancozeb: 0.47 ± 0.08 (*P* < 0.05 versus control group); resveratrol (Low): 0.69 ± 0.04 (*P* < 0.05 versus mancozeb group); resveratrol (High): 0.68 ± 0.09 (*P* > 0.05 versus mancozeb group)).

## DISCUSSION

Toxic mancozeb and its derivative are supposed to accumulate in environment, expose to human and animals from food and drinking water, and exert toxic effects on female reproduction systems. Previous studies have associated mancozeb with decreased female reproductive competency, with the evidence of increase in the number of atretic follicles [31], a general impairment of estrus cycle [32] , pathological changes in the gonads and uterus [14], and disruption of oocyte maturation [9]. In this study, we investigated more details about the toxic effects of mancozeb on reproductive performance via apoptotic relative events. We also reported an anti-oxidant resveratrol could protect mancozeb induced toxicity, regarding to both ovaries and oocytes.

We first observed that resveratrol could block mancozeb induced suboptimal fetal and neonatal outcomes. Decreased litter size and weight occurred in mancozeb treated mice. More notably, some litters showed stillbirth, along with neonatal purpura. These observations may be linked to the suboptimal follicle in ovaries. Indeed, previous reports showed a significant decrease in the number of healthy follicles with concomitant increase in the number of the atretic ones after mancozeb treatment [8]. Besides, decreased weight of ovary and increased number of corpora lutea were also associated with mancozeb [33]. All of these are in accordance with our results in this study. In fact, mechanistically related these phenomenal are associated with apoptosis of granulosa cells and oocytes, and mancozeb could aggravate this process [34, 35]. We thought resveratrol can block mancozeb induced apoptosis and considered it as the essence of the protective effect of resveratrol to the mancozeb induced toxicity on female fertility.

It is well known that oocyte meiotic maturation and fertilization are two crucial events in female reproduction. During this process, mammalian oocytes undergo two rounds of asymmetric divisions, generating a large haploid cell called oocyte. In our study, no differences were observed in polar body formation between control and mancozeb treated group; however, a decreased formation of pronuclear and 2-cell in the mancozeb treated group were found. These observations are consistent with previous findings obtained in adult female rats exposed mancozeb for 30 days, whose oocytes showed a decrease fertilizing potential [10]. In the oocytes, the cytoskeleton including microtubules and actin microfilament is essential for the cytokinesis and it is involved in cortical reorganization and spindle assembly [36]. Microtubules make up the meiotic spindle promoting chromosome alignment and segregation, while actin filaments adjust meiotic spindle migration and initiate cytokinesis for polar body extrusion [37]. This prompt us to check whether resveratrol could counteract the mancozeb induced cytotoxicity on oocytes through the improvement of cytoskeleton dynamics. Indeed, resveratrol could alleviate the decreased accumulation of actin filament towards the cortex in the mancozeb group, together with a higher incidence of central spindle position. Furthermore, microfilament nucleation factor Skap2 expression was improved by resveratrol. These results suggested that resveratrol could partially protect oocyte from mancozeb induced loss of polarity, allowing the cytokinesis to proceed without any abnormality.

We next confirmed mancozeb induced accumulations of ROS in mouse oocytes, which was consistent with the observed CAT and SOD increase. These observations underlined that mancozeb induced oxidative stress in mouse oocytes. Accumulation of ROS is known to interfere with nuclear and cytoplasmic maturation and may lead to cell death [38]. Some reports confirmed this ROS, in human mesencephalic cells and mouse granulosa cells, after mancozed treatment, since the level of oxidative stress related gene HSPA1A, HSPA1B, NOX5, SOD2 and CAT were up-regulated, leading to oxidative stress response [7]. In addition, anti-oxidant supplement would protect from mancozeb induced toxicity [39, 40], suggesting and confirming that mancozed toxicity is not only due to its estrogen-like effects but also resulting from the cell damage related to oxidative stress. Resveratrol improved mouse oocyte antioxidant system, which has been suggested previously [41] . As a polyphenol, resveratrol can scavenge ROS with its phenolic hydroxyl group in oocytes. Besides, resveratrol could also protect embryo from nicotine induced oxidative stress [42]. In this case, resveratrol protected oocytes from mancozeb increased ROS, with the evidence of decreased fluorescence intensity of DCFH-DA, suggesting an improved condition for oocyte development formed, indirectly preventing subsequent mancozeb induced apoptosis.

Indeed, apoptosis plays a major role in oogenesis and ovulation [43]. This nomenclature committee on cell death (NCCD) process is regulated tightly; and its mechanism involves mainly two alternative signaling pathways: death receptor-mediated “extrinsic apoptotic pathway” and the mitochondrion-mediated “intrinsic apoptotic pathway” which occurs in oocytes and granulosa cells [44]. Previous studies showed that mancozeb can exert its pro-apoptotic ability on lymphocytes [45], mononuclear cells [46] and neurocytes [47]. More notably, mouse granulosa cells exposed to mancozeb underwent a time- and dose-dependent modification of apoptotic features [12]. In this study, we indeed observed apoptosis in both granulosa cells and oocytes, while, resveratrol could reduce the frequency of the apoptotic events. These results demonstrated that resveratrol could exert an anti-apoptotic function, thus alleviating mancozeb induced ovarian and oocyte toxicity.

The activity and organization of mitochondria are important features among diverse events involved in cytoplasmic maturation. Unlike other somatic cells, the lack of glycolytic enzyme system in oocytes makes the oxidative phosphorylation pathway in mitochondria the only source of ATP for the entire energy required by all cellular activities. The mitochondrial distribution pattern is a highly dynamic process during oocyte maturation and early embryonic development [48]. Consistent results revealed that a suboptimal mitochondria distribution in the ooplasm is a marker of cytoplasm immaturity and is strongly related to a decreased developmental ability [44, 49]. Besides, a characteristic of mitochondrion-mediated “intrinsic apoptotic pathway” is the mitochondrial membrane potential depolarization, which simultaneously disturb mitochondrial respiration and functional correct electron transportation. A previous research has associated inefficient mitochondrial biogenesis concomitant with decreased developmental competence of IVM oocytes and embryos [50]. Here, we shows that resveratrol played an importantly role in maintaining mitochondrial homeostasis, acting in both their function and distribution. Taking into account the relationship among mitochondrial function, apoptosis, and energy requirements for various key events in the oocyte maturation, the reduced alterations of mitochondrial morphology induced by mancozed that we detected, might indicate that resveratrol could reduce the mancozed adverse effects on electron transfer and mitochondrial distribution, subsequent ROS accumulation and apoptosis activation.

Specific histone modification patterns are present during oocyte maturation and may play specific roles in meiosis. Previous studies demonstrated that increased epigenetic modifications are related with acquired meiotic and developmental potential during oocyte maturation. Varies of methylation patterns of H3 histones during murine oocyte meiotic maturation also has been documented. Indeed, unfinished modification of lysine residue 4 of histone H3 (H3K4me) due to methylation failure occurred in the oocytes of ageing individuals [51]. A suboptimal H3K9 methylation also decreased the full developmental competence of mouse oocytes [52]. Our results showed that resveratrol and mancozeb exposure affected the levels of H3K4me2 and H3K9me2, suggesting that resveratrol protective effect might be exerted through correcting against the changed in H3 histones methylation status induced by mancozeb. The methylation of lysine residue 27 of histones H3 undergo a dramatic alteration during the oocyte maturation and embryo development [53]. Besides, a correlation between H3K27 methylation and apoptosis has been discovered. Previous study demonstrated that down-regulation of methyltransferase EZH2 may induce apoptosis in colon cancer cells [54], which is consistent with our results showing that mancozeb increased H3K27 methylation levels and induced apoptosis in oocytes. Therefore, it is reasonable to propose that the positive trend of histone modification modes changed by resveratrol remedied the toxic effect of mancozeb and increased the oocyte developmental potential.

Previous studies showed that birth defects are closely related to the mother exposure to mancozeb. In the present study, we showed that the reproductive toxicity is involved in ROS mediated apoptosis and epigenetic modification. We also find resveratrol could play an important role in promoting ovary and oocyte quality by interfering mancozeb induced accumulations of ROS, abnormal mitochondrial function, increased apoptotic tendency, decreased development potential of oocytes, and epigenetic modifications, collectively, improve the reproductive outcomes. Future work should concentrate on the in-depth mechanism of the protective effects of resveratrol on the mancozeb induced damage such as the alteration of protein expression profile and cellular signal transduction.

## MATERIALS AND METHODS

### Ethic statement and mice feeding

Care and handing of 4–6 week-old ICR mice were conducted in strict accordance with the guidelines of Xiamen University Animal Studies Committee, China (approval ID: XMUMC 2011-10-08). Two-hundred and forty ICR mice were maintained on *ad libitum* access to water and standard murine chow diet under a 12–12 h light-dark cycle. Mice were randomly divided into four groups (*n* = 60/group): mancozed, mancozed+ resveratrol (Low), mancozed+ resveratrol (High) and control. The three mancozed groups received an oral administration at a dose of 800 mg/kg/day, according to previous studies [33]. The two resveratrol groups received resveratrol in water supplemented at a dose of 100 mg/l (Low) and 200 mg/l (High), according to previous studies [41]. Resveratrol stock solution was stored at 4°C in the dark. Sterile drinking water with resveratrol in bottles covered with aluminum was freshly prepared and provided every week.

### Histological evaluation of ovarian follicles

Ovaries were randomly collected from each different group. After fixeation with 4% paraformaldehyde for 24 h, tissues were embedded in paraffin wax. Serial sections (5 μm) from each ovary were aligned in order on glass microscope slides, and stained with hematoxylin and eosin Y (HE). Scanning of ovarian sections under dot slide-digital virtual microscope was performed according to standard procedures.

### Oocyte collection and culture

Mice were given 10 IU pregnant mare serum gonadotropin (PMSG; Ningbo Sansheng Pharmaceutical Company, China) intraperitoneally. After 48 h, the mice were killed and ovaries were removed. GV-intact oocytes were collected in M2 medium (Sigma, USA) and were cultured under paraffin oil in a 5% CO2, 95% air incubator. Oocytes to be blocked at the GV stage were cultured in M2 medium plus 2.5 mM milrinone (Cayman, USA), whereas those allowed to mature *in vitro* were cultured in fresh M2 medium for 8.5 or 12.5 hr. For parthenogenesis, *in vitro* matured MII oocytes were activated with 10 μM SrCl2 for 4–6 hours. The oocytes were then observed with light microscopy to observe the pronucleus; the activated eggs were then cultured in the KSOM medium to the 2-cell embryo stage.

### Immunofluorescence microscopy

The protocol was basically the same as described in our previous studies [49, 55]. Briefy, oocytes were fixed with 4% paraformaldehyde at room temperature for 30 min and then transferred to a permeabilization solution (0.5% Triton X-100) for 30 min. After 1 h in 1% bull serum albumin (BSA)-supplemented PBS, oocytes were incubated at 4°C overnight with the following primary antibodies: 1:100 rabbit polyclonal anti-Di-methyl-Histone H3 (Lys9) (H3K9me2) (Abcam, UK), 1:100 Rabbit monoclonal anti-Di-methyl-Histone H3 (Lys27) (H3K27me2) (Cell Signaling Technology, USA), 1:100 rabbit polyclonal anti-Di-methyl-Histone H3 (Lys4) (H3K4me2) (Cell Signaling Technology, USA), 1:200 mouse anti-α-tubulin-FITC antibody (Sigma, USA) or 1:100 Phalloidin-TRITC (Sigma, USA). After three washes in PBS containing 0.1% Tween 20 and 0.01% Triton X-100 for 5 min each, the oocytes were labeled with specific fluorescence secondary antibodies. Finally, oocytes were co-stained with DAPI (Vector, Switzerland), mounted on glass slides with mounting medium, and slices were examined using FV1000 confocal laser scanning microscope (Olympus, Japan).

To determine ROS production, mitochondrial distribution and oocytes apoptosis, dichlorofluorescein diacetate (DCFH-DA) (Beyotime, China), MitoTracker Red (Invitrogen, USA) and Annexin-V probe (Beyotime, China) were respectively used according to the manufacturer's instruction. Briefly, live oocytes were cultured in M2 medium plus specific probe for 30 min at 37°C, 5% CO_2_ air. After washing, stained oocytes were fixed and analyzed with the FV1000 confocal laser scanning microscope.

To evaluate apoptosis, fixed ovary slices were treated with TUNEL (terminal deoxynucleotidyltransferase-mediated dUTP nick-end labelling) reaction mixture (Keygene biotech, China), counterstained with DAPI and mounted in mounting medium. TUNEL-positive and total cell nuclei were observed with the FV1000 confocal laser scanning microscope.

### Gene expression by real-time PCR

Gene expression was determined by real-time quantitative PCR and calculations were performed using the ΔΔCT method. Total RNA was isolated from 80 oocytes using Dynabeads mRNA DIRECT kit (Invitrogen, USA), and subjected to cDNA synthesis with a cDNA synthesis kit using Oligo (dT) 12–18 primers (Takara, Japan). SOD, GSH-Px, ASH1L, SETDB1 and EZH2 were amplified using the specific primers (Sangon, China) (Supplementary Table S1). SYBR Green Real-time PCR Master Mix kit (Toyobo, Japan) was used with a Step One Real-time PCR System (Applied Biosystems, USA) under the following conditions: 95°C for 10 min, 40 cycles of 95°C for 15 s, and 60°C for 1 min.

### Immunoblot analysis

A total of 180 mouse oocytes were lysed in 4 × loading buffer and heated at 100°C for 5 min. Proteins were separated by sodium dodecyl sulfate polyacrylamide gel electrophoresis (SDS-PAGE) and then electrophoretically transferred into nitrocellulose membranes. Membranes were blocked with 5% bovine serum albumin and then incubated with primary antibodies overnight at 4°C. After washing three times in PBST (10 min each), membranes were incubated with secondary antibody for 1 h at room temperature. Finally, membrane bands were detected by Western Lightning ECL profession detection reagent (Keygene biotech, China).

### Statistical analysis

At least three biological replicates were used for each treatment. Data were expressed as mean ± SEM and were analyzed by ANOVA using SPSS software (IBM Corp, USA) followed by the Student-Newman-Keuls test. Difference at *P* < 0.05 was considered statistically significant.

## SUPPLEMENTARY MATERIALS TABLE


