# Supplementary material for: Protective effects of resveratrol against mancozeb induced apoptosis damage in mouse oocytes
Source: Oncotarget. 2016 Dec 21;8(4):6233–45. doi: 10.18632/oncotarget.14056 (PMC5351627; doi:10.18632/oncotarget.14056)
Supplement: Supplementary file 1 [file oncotarget-08-6233-s001.pdf]

Protective effects of resveratrol against mancozeb induced apoptosis damage in mice oocytes

Supplementary Materials

Supplementary Table S1: Primer sequence

Primer name	Primer sequence	
	Forward	Reverse
SOD	5′-aaagcggtgtgcgtgctgaa-3′	5′-caggtctccaacatgcctct-3′
GSH-px	5′-cctcaagtacgtccgacctg-3′	5′-caatgtcgttgcggcacacc-3′
ASH1L	5′-gctacctgtcctccttcc-3′	5′-aacccaactgctgtgcta-3′
SETDB1	5′-ttggcaaagtactcatcaccca-3′	5′-ttggatgacattgccaaaggct-3′
EZH2	5′-cagataagggcaccgcagaa-3′	5′-acattcaggaggcagagcac-3′
